# Renin-angiotensin system inhibition in COVID-19 patients

**DOI:** 10.1007/s12471-020-01439-5

**Published:** 2020-06-08

**Authors:** A. A. F. de Vries

**Affiliations:** Laboratory of Experimental Cardiology, Department of Cardiology, Leiden University Medical Centre, Leiden, The Netherlands

**Keywords:** Angiotensin-converting enzyme, Angiotensin-converting enzyme inhibitor, Angiotensin II type‑1 receptor blocker, Coronavirus disease 2019, Renin-angiotensin system, Severe acute respiratory syndrome coronavirus 2

## Abstract

Angiotensin-converting enzyme (ACE) inhibitors (ACEIs) and angiotensin II type‑1 receptor blockers (ARBs) are among the most widely prescribed drugs for the treatment of arterial hypertension, heart failure and chronic kidney disease. A number of studies, mainly in animals and not involving the lungs, have indicated that these drugs can increase expression of angiotensin-converting enzyme 2 (ACE2). ACE2 is the cell entry receptor of severe acute respiratory syndrome coronavirus 2 (SARS-CoV-2), the causative agent of coronavirus disease 2019 (COVID-19) that is currently battering the globe. This has led to the hypothesis that use of ACEIs and ARBs may increase the risk of developing severe COVID-19. In this point of view paper, possible scenarios regarding the impact of ACEI/ARB pharmacotherapy on COVID-19 are discussed in relation to the currently available evidence. Although further research on the influence of blood-pressure-lowering drugs, including those not targeting the renin-angiotensin system, is warranted, there are presently no compelling clinical data showing that ACEIs and ARBs increase the likelihood of contracting COVID-19 or worsen the outcome of SARS-CoV‑2 infections. Thus, unless contraindicated, use of ACEIs/ARBs in COVID-19 patients should be continued in line with the recent recommendations of medical societies.

In late December 2019, several clusters of pneumonia cases of unknown aetiology were reported in the city of Wuhan, People’s Republic of China. Investigation of respiratory samples of these cases identified a novel coronavirus (CoV) as the causative agent [1, 2]. Nucleotide sequence analysis of the viral RNA genome revealed it to be closely related to that of the betacoronavirus responsible for the outbreak of severe acute respiratory syndrome (SARS) in Asia Pacific (and Canada) in 2002–2003. From China, the new virus, which was designated SARS-CoV‑2, has rapidly spread throughout the world with a total of 5,697,334 confirmed cases and 355,758 deaths as of 28 May 2020 [3]. These figures are likely a gross underestimation of the actual impact of SARS-CoV‑2 due to limited virological testing and underreporting. The primary routes of SARS-CoV‑2 transmission are respiratory droplets and direct contact. Approximately 80% of SARS-CoV‑2 infections are relatively mild (with flu-like symptoms) or even asymptomatic. Some 15% of cases result in severe disease (so-called COVID-19) characterised by pneumonia and dyspnoea, while ~5% of SARS-CoV-2-infected individuals experience critical illness (i.e. acute respiratory distress syndrome (ARDS), septic shock, (multi‑)organ failure) and require intensive care.

Like the original SARS-CoV, host cell penetration by SARS-CoV‑2 relies on the interaction of the viral spike (S) protein with angiotensin-converting enzyme 2 (ACE2) [4]. ACE2 is a monocarboxypeptidase present on the surface of a wide variety of different cell types, including epithelial cells lining the respiratory tract, cardiac fibroblasts, cardiomyocytes, endothelial cells and vascular smooth muscle cells (VSMCs) [5, 6]. In the lungs, ACE2 expression is mainly found in alveolar macrophages and in the surfactant-producing type II pneumocytes and to a lesser extent in bronchial and tracheal epithelial cells [5]. ACE2 is a paralogue of angiotensin-converting enzyme (ACE). Both these enzymes are Zn^2+^-dependent transmembrane proteins involved in the production of vasoactive peptides [7, 8]. However, ACE and ACE2 generally have opposite effects, thus functioning as counter-regulatory factors within the renin-angiotensin system (RAS).

ACE converts angiotensin I (AngI/angiotensin-(1–10)) into angiotensin II (AngII/angiotensin-(1–8)) (Fig. 1). Binding of AngII to the AngII type‑1 receptor (AT1R) has pro-inflammatory, pro-oxidative, pro-apoptotic and pro-fibrotic effects, increases vascular tone and leakage (Fig. 1) and is involved in pathophysiology of many different tissues and organs [9]. Stimulation of AT1R on the surface of VSMCs by AngII results in the activation of signalling pathways that promote VSMC contraction. Excessive AngII-AT1R signalling also induces the proliferation, migration and growth of VSMCs, promotes vascular remodelling and contributes to initiation and progression of atherosclerosis by inducing endothelial dysfunction [9]. Activation of AT1Rs in cardiac myocytes induces cellular hypertrophy, while binding of AngII to AT1Rs on the surface of cardiac fibroblasts results in cardiac fibrosis by stimulating the synthesis of extracellular matrix proteins, including collagen type I and III, and by inducing proliferation and migration of cardiac fibroblasts [9]. Unbalanced AT1R signalling in the lungs is associated with airway inflammation, bronchial hyper-responsiveness, fibrosis and pulmonary hypertension. AngII is also an important driving force in the inflammatory cascade and alveolar epithelial injury associated with ARDS [10, 11]. Apart from binding to AT1R, AngII can also bind to the Ang II type‑2 receptor (AT2R) (Fig. 1). Stimulation of this receptor has effects that are largely opposite to those induced by AngII-AT1R signalling. Under pathophysiological conditions, AngII-AT1R signalling is generally dominant over AngII-AT2R signalling.

**Fig. 1:**
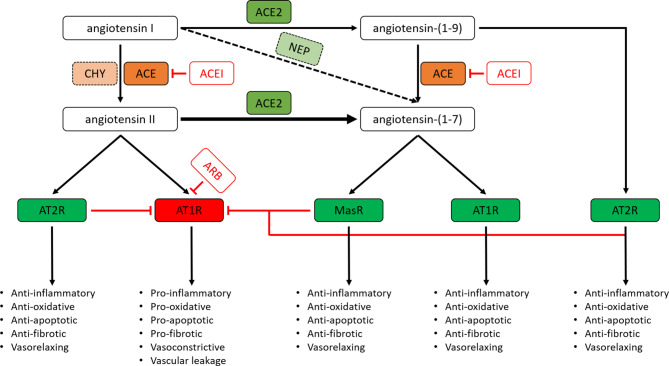
Overview of major ACE and ACE2 substrates and products, the G‑protein-coupled receptors (GPCRs) activated by these products, the biological consequences of the stimulation of these GPCRs and the mode of action of ACE inhibitors (*ACEI*) and angiotensin II type‑1 receptor blockers (*ARB*). Besides ACE and ACE2, other proteolytic enzymes are also involved in the processing of renin-angiotensin system components, e.g. AngII can also be generated from AngI by chymase (*CHY*; secreted by mast cells) and Ang1–7 can be produced from AngI by neprilysin (also known as neutral endopeptidase (*NEP*)) [8, 13]

ACE2 can cleave AngI to generate angiotensin-(1–9) (Ang1–9) and AngII to produce angiotensin-(1–7) (Ang1–7) (Fig. 1). The conversion of AngII into Ang1–7 by ACE2 not only reduces detrimental AngII/AT1R signalling but also generates the ligand for the Mas receptor (MasR), activation of which opposes the effects of AT1R stimulation by AngII (Fig. 1). Recently, Ang1–7 was shown also to exert beneficial effects by binding to the AT1R and inducing β‑arrestin-biased signalling through this receptor [12]. Ang1–9 is another ACE2 reaction product involved in counterbalancing AngII/AT1R signalling by stimulating AT2R signalling (Fig. 1).

The recognition of the adverse effects of unopposed AngII-AT1R signalling led to the development of ACE inhibitors (ACEIs) and AT1R blockers (ARBs). As shown in Fig. 1, ACEIs inhibit the production of AngI and thereby indirectly prevent harmful AT1R signalling. Analysis of plasma vasoactive peptide levels has shown a moderate decrease in AngII and a large increase in Ang1–7 (e.g. generated from AngI by neprilysin) following ACEI treatment [8], Thus, ACEIs not only suppress detrimental AngII-AT1R signalling but also promote beneficial Ang1–7 signalling. ARBs, on the other hand, do not block AngII production but selectively and competitively inhibit the binding of AngII to AT1R (Fig. 1). The blood plasma of patients receiving ARBs contains strongly elevated AngII and moderately increased Ang1–7 levels [8].

Due to their well-established anti-hypertensive, anti-oxidative, anti-inflammatory, anti-fibrotic and anti-hypertrophic effects, ACEIs and ARBs have found widespread use in clinical practice. As a consequence, a large percentage of cardiovascular patients, including those suffering from systolic heart failure (HFrEF) receive ACEIs or ARBs to lower their blood pressure by reducing systemic vascular resistance (and to slow down adverse cardiac remodelling). In Europe, ACEIs are part of the first-line treatment of (a)symptomatic HFrEF patients unless contraindicated or not tolerated [14]. In the latter case, ACEIs are commonly replaced by ARBs. In the United States either ACEIs or ARBs are recommended in the first-line pharmacological treatment of HFrEF [15]. The European Society of Cardiology guidelines recommend the use of AT1R-neprilysin inhibitors (ARNIs) instead of ACEIs or ARBs when patients remain symptomatic in spite of optimal treatment with ACEIs/ARBs, beta-adrenergic receptor antagonists and mineralocorticoid receptor blockers. The guidelines of the American College of Cardiology and American Heart Association recommend the prescription of ARNIs to chronic symptomatic NYHA class II and III HFrEF patients who tolerate ACEIs/ARBs to further reduce morbidity and mortality. The replacement of ACEIs/ARBs by ARNIs is supported by a recent meta-analysis showing that ARNIs are superior to ACEIs and ARBs in mediating reverse cardiac remodelling in HFrEF patients [16].

By analogy to what happens with SARS-CoV, it is widely assumed that infection of (airway epithelial) cells results in a strong reduction of ACE2 expression at the plasma membrane but does not affect ACE (surface) levels [17]. In the same study, the ARB losartan attenuated SARS-CoV S‑protein-induced lung injury in a mouse model. Moreover, serum AngII levels are significantly elevated in COVID-19 patients and exhibit a linear positive correlation to viral load and lung injury as determined by oxygenation index [18]. This led to the hypothesis (hypothesis 1) that SARS-CoV-2-mediated downregulation of ACE2 disturbs the balance between ACE/AngII/AT1R and ACE2/Ang1–7/MasR signalling in the lungs and thereby contributes to the development of ARDS in COVID-19 patients (Fig. 2). If so, ACEIs and ARBs may have a beneficial effect on the course of COVID-19 by reducing ACE/AngII/AT1R signalling and thereby restoring the local balance between the activities of the ACE/AngII/AT1R and ACE2/Ang1–7/MasR axes (Fig. 3). Support for this idea comes from a study by Henry et al. showing favourable effects of ACEI treatment during hospitalisation of patients with (non-corona)viral pneumonia [19]. Also, several research groups have demonstrated that ACEIs and ARBs can (1) attenuate the lung injury caused by (experimental) ARDS and (2) reduce pulmonary arterial hypertension [10, 11].

**Fig. 2:**
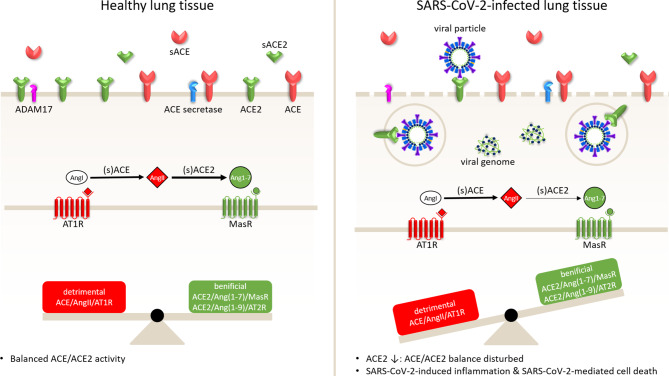
SARS-CoV‑2 uptake by receptor-mediated endocytosis directly and indirectly lowers ACE2 surface expression, reduces conversion of AngII into Ang1–7 and hence causes a misbalance in the activities of the ACE/AngII/AT1R and ACE2/Ang1–7/MasR axes. This, together with SARS-CoV-2-induced cell death, inflammation and thromboembolism, is responsible for the severe lung injury seen in critically ill COVID-19 patients. A disintegrin and metalloprotease 17 (*ADAM17*) and ACE secretase are membrane-bound proteases involved in the production, by enzymatic cleavage, of soluble (*s*) ACE2 and ACE, respectively. AngII-AT1R signalling increases ADAM17 expression, leading to a reduction in surface ACE2 levels due to sACE shedding

**Fig. 3:**
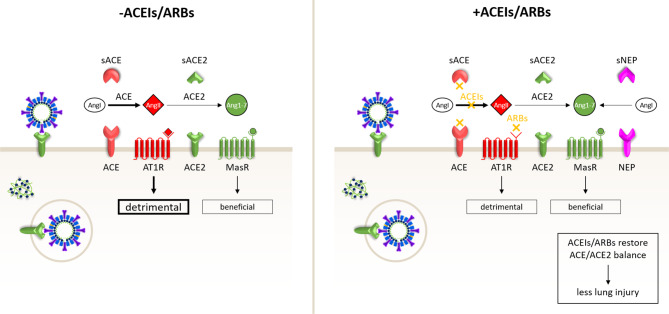
Schematic representation of hypothesis 1. ACE inhibitors (*ACEIs*) and angiotensin II type‑1 receptor blockers (*ARBs*) alleviate COVID-19 by inhibiting the harmful ACE/AngII/AT1R axis, which gained dominance due to SARS-CoV-2-induced downregulation of ACE2

A number of scientists [20] has raised the opposite idea (hypothesis 2) that ACEI and ARB pharmacotherapy may aggravate SARS-CoV-2-induced lung disease by increasing ACE2 surface expression on airway epithelial cells (Fig. 4). Several animal studies have indicated an increase of ACE2 expression following treatment with ACEIs or ARBs although, especially for ACEIs, decreases or no alterations in ACE2 expression have also been reported (reviewed in [21]). Information on the effect of these anti-hypertensive drugs in humans is limited to the analysis of duodenal biopsies. Collectively, the data show a more consistent upregulation of ACE2 by ARBs than by ACEs [21] in various tissues. However, whether ACE2 expression is upregulated by either of these drugs in the primary target cells of SARS-CoV‑2 in human lungs is not yet known.

**Fig. 4:**
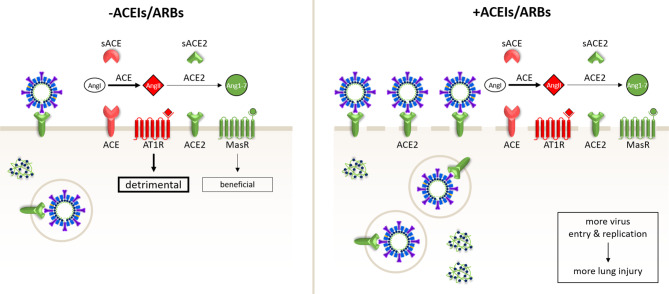
Schematic representation of hypothesis 2. ACE inhibitors (*ACEIs*) and angiotensin II type‑1 receptor blockers (*ARBs*) aggravate COVID-19 by upregulating ACE2, causing increased SARS-CoV‑2 entry and replication

The hypotheses presented above are not mutually exclusive. In patients that are on ACEIs/ARBs before infection with SARS-CoV‑2, ACEI/ARB-mediated upregulation of ACE2 in airway epithelium could increase the risk of becoming infected with SARS-CoV‑2 and may facilitate initial virus propagation/spread. In patients that receive ACEIs/ARBs once they are infected with SARS-CoV‑2, these drugs could mitigate the extent of lung injury by inhibiting detrimental AngII/AT1R signalling and hence restoring the balance with beneficial Ang1–7/MasR signalling.

Recently, a number of observational studies were performed to investigate the influence of ACEI and ARB pharmacotherapy on SARS-CoV‑2 infections [22–28]. None of the studies showed use of these anti-hypertensive/cardioprotective drugs to be associated with an increased risk of (1) SARS-CoV‑2 infection, (2) severe COVID-19 or (3) SARS-CoV-2-related in-hospital death. Conversely, in the small-scale study (205 patients) of Bean and colleagues, a lower rate of death or transfer to a critical care unit within 7 days was found in patients on an ACE-inhibitor [22]. Likewise, in the study of Mehra and co-workers involving 8910 COVID-19 patients, the use of ACEIs was associated with better survival [25]. Moreover, in the small-scale studies of Meng et al. [26] and Zhang et al. [28], favourable effects of ACEIs/ARBs on, respectively, severity of disease and all-cause mortality were observed. Although each of these studies arrives at the same conclusion, namely that ACE/ARB pharmacotherapy is unlikely to be harmful for COVID-19 patients (and may even have beneficial effects), small-scale studies have a high risk of chance associations and with observational studies there is always the possibility of confounding. Because of this and some other weaknesses (e.g. use of different virological tests within one study, lack of information about the continuation of ACE/ARB treatment during hospitalisation, bias resulting from selective virological testing), randomised controlled trials will be required to definitively answer the question whether RAS inhibitors or other blood-pressure-lowering drugs (e.g. ARNIs, beta blockers, calcium channel blockers, thiazide diuretics) are harmful to COVID-19 patients. Ideally, these trials should also investigate the effect of different combinations of anti-hypertensive drugs and the timing of anti-hypertensive drug administration on the course of COVID-19. Of special interest is the effect of ARNIs on COVID-19, as these drugs cause a large rise in plasma AngII levels but do not increase the concentration of Ang1–7 in blood plasma [8].

Apart from further exploring the possible benefit of chemical renin-angiotensin-aldosterone system inhibitors in COVID-19 patients, another promising approach might consist of the treatment of COVID-19 with human recombinant soluble ACE2. This biological drug could inhibit virus entry into host cells by competing with the binding of the S protein to membrane-bound ACE2 and at the same time increase Ang1–7 levels at the expense of AngII.
